# Is relatively young age within a school year a risk factor for mental health problems and poor school performance? A population-based cross-sectional study of adolescents in Oslo, Norway

**DOI:** 10.1186/1471-2458-5-102

**Published:** 2005-10-05

**Authors:** Lars Lien, Kristian Tambs, Brit Oppedal, Sonja Heyerdahl, Espen Bjertness

**Affiliations:** 1 Institute of General Practice and Community Medicine, University of Oslo, PO Box 1130, Blindern, Oslo 0318, Norway; 2 The Norwegian Public Health Institute, PO Box 4404 Nydalen, 0403 Oslo, Norway; 3 Regional Centre for Child and Adolescent Psychiatry, Region East and South, 23 Tasen, 0801 Oslo, Norway

## Abstract

**Background:**

Several studies have shown that children who are relatively young within a school year are at greater risk for poorer school performance compared with their older peers. One study also reported that relative age within a school year is an independent risk factor for emotional and behavioral problems. The objective of this study was to test the hypothesis that relatively younger adolescents in the multiethnic population of Oslo have poorer school performance and more mental health problems than their relatively older classmates within the same school year.

**Methods:**

This population-based cross-sectional study included all 10^th^-grade pupils enrolled in 2000 and 2001 in the city of Oslo. The participation rate was 88%. Of the 6,752 pupils in the study sample, 25% had a non-Norwegian background. Mental health problems were quantified using the abbreviated versions of Symptom Check List-25 (SCL-10) and the Strength and Difficulties Questionnaire (SDQ). Information on school performances and mental health problems were self-reported. We controlled for confounding factors including parental educational level, social support, gender, and ethnicity.

**Results:**

The youngest one-third of pupils had significantly lower average school grades than the middle one-third and oldest one-third of their classmates (p < 0.001). Of the mental health problems identified in the questionnaires, the groups differed only on peer problems; the youngest one-third reported significantly more problems than the middle and oldest groups (p < 0.05). Age within a school year and gender showed significant interactions with total SDQ score, SDQ peer problems score, SDQ pro social score, and SCL-10 score. After stratifying for gender, the peer problem scores differed significantly between age groups only among boys. The SCL-10 score was significant, but only in girls and in the opposite direction to that expected, with the oldest pupils having significantly higher scores than the other two groups (p < 0.05).

**Conclusion:**

In adolescents from a multicultural city in Norway, relative age within a school year significantly influenced academic performance. In contrast to data from Great Britain, relative age within a school year was not an important risk factor for mental health problems in adolescents in Oslo.

## Background

Teachers and parents claim that the youngest pupils in school classes perform less well and have more conduct and hyperactivity problems than their older classmates [1]. These allegations have been confirmed in several studies of school performance [2], special educational needs [3,4], learning difficulties [5], and academic performance [6]. Different theories have been proposed to explain this effect. One explanation is that a season-of-birth effect is linked to prenatal exposure to infections [7], similar to the effect identified for schizophrenia and major depression [8]. Another rationalization is that pupils who are relatively younger have less preschool experience and are therefore less well equipped to meet the demanding expectations at school [9]. A third explanation is the age-position effect, which maintains that the oldest are more socially advantaged, mature, and satisfied at school than relatively younger pupils within the same school year [5].

The explanation of a biological season-of-birth effect has been discounted [5] since the phenomenon of poorer performance in relatively younger pupils seems to occur irrespective of different cut-off dates for school enrolment in different countries [10] and geographical regions [11]. The poorer performance in relatively younger pupils is more likely to be related to limited preschool experience or an age-position effect. In Norway and most western countries where all children born in the same calendar year enter school at the same time, the oldest and youngest pupils may differ in age by nearly one full year.

A recent study reported that the effect of relative age of entry into school is also an independent risk factor for mental health problems [11]. The study population was in England, Wales, and Scotland, which have different cut-off dates for school entry: 1 September for England and Wales, and 1 March for Scotland. Children born in summer (May-August) in England and Wales, and in winter (November-February) in Scotland were disadvantaged. The study included the self-report part of the Strength and Difficulties Questionnaire (SDQ) for the age group 11–15 years. Our study was based on the same questionnaire and a similar age group, making these studies directly comparable.

Parental educational level is strongly related to their children's school performance, and is associated with mental health problems [12-14]. A recent study from Norway showed that social support is one of the psychosocial variables that have proven to be consistently associated with mental health during adolescence [15]. Even if research studies have demonstrated that minority youth report the same level of mental health as their majority peers, some groups seems to be at increased risk of mental health problems [16,17]. Several studies has found that girls perform better than boys at school, and that girls have more mental health problems [3,18]. Even if these factors do not relate systematically to seasonal birth, such a relationship cannot be completely ruled out, and the effect of children's ages at school entry should be adjusted for these factors. We controlled for these factors, and examined the possible interaction effects of ethnicity, gender, and age at entry into school on our main outcome variables.

The objective of this study was to explore, in a multicultural population, the association between relative age at school entry, school performance, and mental health problems in Norwegian adolescents. We hypothesized that pupils who are relatively younger when they enter school have lower average school grades and more mental health problems than their older classmates. We controlled for parental educational level, ethnicity, and perceived social support. We designed our study to be similar to other studies to compare our data with those of previous studies in this area.

## Methods

### Sample

The Oslo Health Study 2000–2001 was conducted as a joint collaboration between the Norwegian Institute of Public Health, the University of Oslo, and the Municipality of Oslo. The part of the study that examined the adolescents in the sample encompassed all pupils in 10^th ^grade (15–16 years old) included in the class lists for each school in Oslo County during the spring terms (March-June) of 2000 and 2001.

All pupils completed two questionnaires during two school sessions. A project assistant was present in the classroom to instruct the students and to perform the practical parts of the survey, such as distributing and collecting the questionnaires. Questionnaires were left at the school for students absent from school on the day of the survey. The school was contacted if the questionnaires were not returned after a while. Students who had not completed the questionnaires after some months were sent questionnaires to complete at home and return in a prepaid return envelope.

A total of 7,343 pupils, representing 88.3% of the 8,316 eligible subjects, answered at least one question. The participation rates were 86.1% in boys and 90.6% in girls. Of the 7,343 pupils, 590 (8.0%) were excluded because they had entered school either one year later or one year earlier than their classmates. The final number of participants whose questionnaires were analyzed was 6,752, comprising two cohorts. The gender, age, and month-of-birth distributions of the participants were representative for the Norwegian population born in 1985–86. All data were self-reported by the pupils.

### Variables

*The 10-item Symptoms Check List (SCL-10) *was used as to measure psychological distress. SCL-10 [19] has approximately the same sensitivity and specificity for detecting psychological symptoms or global distress as the more widely used SCL-25 [20,21] and correlates highly (r = 0.97) with the 25-item version [22]. The 10 items included in the short version are:

Suddenly scared for no reason

Feeling fearful

Faintness, dizziness, or weakness

Feeling tense or keyed up

Blaming yourself for things

Difficult falling asleep, staying asleep

Feeling hopelessness about the future

Feeling blue

Feeling everything is an effort

Feeling of worthlessness

Each item is rated on a scale from 1 (not at all) to 4 (extremely). The distribution of the SCL-10 data for our participants was highly skewed, and the data were transformed logarithmically to approximate a normal distribution.

*The Strength and Difficulties Questionnaire (SDQ) *is a recently developed questionnaire to assess mental health problems in children and adolescents aged 4–16 years. Its reliability and validity are generally satisfactory [23,24]. The questionnaire has 25 items and scores are classified into five subscales: emotional symptoms, conduct problems, hyperactivity, peer problems, and prosocial behavior. The first four subscales are summed to give the total difficulties score. Each of the SDQ items was scored 1 to 3, with the options "not correct", "partly correct", and "completely correct".

#### Average grade

The participants were asked to fill in the most recent grade recorded in their school record book in Mathematics, Written Norwegian, English, and Social Science. An average grade score was calculated from these four grades.

#### Season of birth

We divided the calendar year into three parts. Pupils born in January to April were labeled the "oldest group"; those born in May to August, the "middle group"; and those born in September to December, the "youngest group". The terms "oldest", "middle" and "youngest" refer to relative age according to the starting cut-off age of school in Norway, which is 1 January.

#### Social support

The questions on social support were formulated as four positive statements on the pupil's perception: (1) of attachment, (2) that his or her opinions were valued, (3) that he or she was helped or supported, and (4) that he or she felt appreciated for each of the social-network items of family, friends, class, and teacher. The scoring alternatives were "completely agree", "partly agree", "partly disagree", and "completely disagree". The scores for each of the social-network variables were summed into one score for each of the items. The four items were then summed into one variable for total social support. The median was used to dichotomize perceived social support into high and low values [25]. Data were missing for 1.5% of the responses for social support from family, 1.4% for support from friends, 3.9% for support from the class, and 4.5% for support from teachers.

#### Parental education level

Statistics Norway registers data on the education levels of all residents in Norway using the Norwegian Educational Standard (NUS) coding system [26]. The registry data were linked to the questionnaire data, providing us with information on the parents' educational levels. Because only a small number of parents had no formal education or only primary education, this group was classified with those parents with secondary schooling as their highest educational level. Two other groups were defined according to their level of university education: less than ("lower university/college") or more than ("higher university/college") four years of university education. Information on parental education levels was missing for 19% of the participants, and these missing values were included as a separate category for this variable in the analyses.

#### Ethnicity

"Norwegian" was defined as having one or two Norwegian parents, and "minority" was defined as both parents born outside Norway. Forty-nine (0.7%) of the pupils did not give any information about their parents' countries of birth.

#### Height and menarche

The questions asked were "How tall are you now?" and "Have you begun to menstruate?", which was answered "yes" or "no".

#### Missing data for the dependent variables

Seventy individuals (1.0%) had missing data for one or more of the SCL-10 items and were excluded from the analysis. For total SDQ scores and SDQ subscores, the proportion of missing values varied from 225 (3.3%) to 239 (3.6%); 439 (6.9%) of the participants did not provide complete information about grades. A larger proportion of pupils classified in the minority group, and more boys than girls, were excluded because of missing data. The proportion of missing data did not vary systematically by month of birth.

### Statistical methods

SPSS for Windows version 11.0 was used in the statistical analysis. Frequencies were observed and cross-table statistics were tested with Pearson's χ^2 ^tests. Analysis of variance (ANOVA) was applied to each of the mental health and grade outcome variables as dependent variables, and age at school entry as an independent variable. To determine whether season of birth was confounded by ethnicity, gender, or parental education level, and to test for their possible interaction effects, these variables were also entered as predictors. Tukey *post hoc *tests were used to locate any significant differences identified in the ANOVA. Pupils with missing data for any of the variables were excluded from the analysis, with the exception of the variable "parental education level". In the analysis of average grades, data from 735 subjects (10.9%) were removed from the multivariate analysis because of missing data, and in the analysis of the other response variables, data from 6.1%–6.5% of subjects were removed because of missing data.

### Ethics

The study protocol was reviewed the Regional Committee for Medical Research Ethics and approved by the Norwegian Data Inspectorate. The study was conducted in full accordance with the World Medical Association Declaration of Helsinki.

## Results

The population characteristics did not differ significantly between the three relative age groups (Table 1). To test for differences in physical maturity levels, we calculated the average height of boys and the number who had not reached menarche among girls (all based on self report). The average height of the boys differed significantly between groups: average height was 176.9 cm (range, 175.2–178.5 cm) in the oldest group, 175.8 cm (175.3–176.3 cm) in the middle group, and 174.5 cm (173.8–175.2 cm) in the youngest group (F = 4.3, p = 0.01). The percentage of girls who had not reached menarche also differed significantly between groups: 1.8% of the girls in the oldest group, 2.0% of the middle group, and 3.9% of the youngest group had not yet reached menarche (Pearson's χ^2^, p = 0.03).

**Table 1:** Population characteristics and social support in different relative age groups within a school year

	**Oldest third **(n = 2219)	**Middle third **(n = 2160)	**Youngest third **(n = 2373)
**Year of birth**			
1984 cohort	45.1	48.1	47.3
1985 cohort	50.8	49.9	49.0
**Gender**			
Female	49.9	51.9	49.9
**Ethnicity**			
Minority groups	22.8	24.4	25.4
**Parental educational level**			
Higher university	16.9	17.7	17.8
Lower university	26.6	26.0	27.9
Secondary	37.4	37.9	36.5
Missing	19.0	18.5	17.8
**Social support**			
Family low	43.8	41.5	41.6
Class low	36.5	36.0	35.5
Teachers low	41.5	41.4	39.2
Friends low	43.1	41.5	40.9

None of the differences between the groups was significant (p > 0.05) by Pearson's χ^2 ^test.

The eight continuous dependent variables were all SDQ scores (conduct problems, hyperactivity, peer problems, emotional symptoms, prosocial behavior, total SDQ score), logarithm-transformed SCL-10 score, and average school grade. Table 2 shows these scores in the three age groups adjusted for social support, ethnicity, gender, and parental education. The SDQ peer problem scores and average grade differed significantly between age groups: the oldest group scored highest on average grade and lowest on SDQ peer problems, and the youngest group scored lowest on average grade and highest on SDQ peer problems. Of the peer problem questions in the SDQ questionnaire, the age groups differed most on the item "being generally liked".

**Table 2:** Adjusted average scores and SD of total SDQ, five SDQ subscores, SCL-10 score, and grade in different relative age groups within a school year

	**Oldest third**		**Middle third**		**Youngest third**		**p**
	Mean	SD	Mean	SD	Mean	SD	

Total SDQ score	9.81	4.85	9.74	4.88	9.78	4.85	0.81
SDQ Emotional	2.64	2.20	2.53	2.18	2.56	2.20	0.44
SDQ Hyperactivity	3.54	2.01	3.62	2.02	3.55	2.03	0.48
SDQ Conduct problems	2.12	1.61	2.15	1.59	2.15	1.63	0.84
SDQ Peer problems	1.49	1.47	1.49	1.51	1.58*	1.53	0.01
SDQ Pro social	7.45	1.80	7.37	1.82	7.35	1.84	0.97
SCL-10 score	0.79	0.51	0.78	0.51	0.76*	0.49	0.03
Average grade	4.01*	0.79	3.95	0.80	3.92	0.79	<0.001

Scores were adjusted for ethnicity, gender, parental education, and social support from family, class, teachers, and friends. * indicates the group that differed significantly from each other by Tukey's *post hoc *test (p = 0.05).

The groups differed significantly on the SCL-10 score, but in a direction opposite to that expected in that the youngest group scored lower than the other groups. The Tukey *post hoc *test showed that the average grade was significantly higher in the oldest group than in the other groups, but that the middle and youngest groups did not differ. The average SDQ peer problem score was significantly higher in the youngest group than in the other two groups (Table 2).

We used ANOVA to examine the interaction between relative age at entry into school and ethnicity, gender, parental education, and social support from family, friends, class, and teachers for each of the dependent variables. The interaction between gender and relative age was significant for total SDQ score (p = 0.04), SDQ peer problems score (p = 0.01), SDQ prosocial score (p = 0.05), and SCL-10 score (p = 0.03).

Because of the significant interaction between gender and age at entry into school, we stratified the data by gender (Table 3). The differences between age groups in the SDQ peer problem scores were significant only in boys, and in the SCL-10 scores only in girls, but in the opposite directions to those hypothesized. Average grade differed between the relative age groups in both boys and girls. The *post hoc *tests showed that SDQ peer problems and average grades differed between the youngest and oldest groups among boys, and that average grades differed significantly between all groups in girls. None of the other response variables differed significantly when analyzed for boys and girls separately.

**Table 3:** Adjusted average scores for total SDQ, five SDQ subscores, SCL-10 score, and grade in boys and girls in different relative age groups within a school year

	**Boys**				**Girls**			
	Oldest	Middle	Youngest	p	Oldest	Middle	Youngest	p

Total SDQ score	9.01	8.90	9.31	0.07	10.61	10.56	10.37	0.35
SDQ Emotional	1.74	1.67	1.77	0.24	3.55	3.35	3.27	0.07
SDQ Hyperactivity	3.43	3.42	3.44	0.95	3.68	3.78	3.61	0.19
SDQ Conduct problems	2.34	2.24	2.32	0.39	1.91	2.01	1.97	0.46
SDQ Peer problems	1.52 *	1.56	1.74 *	<0.001	1.47	1.42	1.42	0.70
SDQ Pro social	6.96	6.81	6.92	0.41	7.90	7.95	7.84	0.46
SCL-10 score	0.64	0.62	0.62	0.62	0.94	0.94	0.89*	0.003
Average grade	3.90 *	3.87	3.82 *	0.009	4.14 *	4.06 *	4.03 *	<0.001

Scores were adjusted for ethnicity, gender, parental education, and social support from family, class, teachers, and friends. * indicates the groups that differed significantly from each other by Tukey's *post hoc *test (p = 0.05).

Effect size was calculated as the difference in average score between the youngest and oldest group divided by the pooled standard deviations for the response variables that differed significantly between groups. The effect size for boys and girls together was 0.06 for SDQ peer problems, 0.11 for average grades, and 0.06 for logarithm-transformed SCL-10 scores. In boys, the effect size was 0.14 for SDQ peer problems and 0.10 for average grade. In girls, the effect size was 0.04 for average grade and 0.09 for logarithm-transformed SCL-10 scores.

## Discussion

This study confirms the data from several other studies showing that children who are relatively young within their school year perform less well than their older classmates [2-6]. The relationship between relative age and school performance seems to be linear, at least for school performance. However, the effect size was small for both boys and girls, according to Cohen's classification [27]. To our knowledge, this is the first study to demonstrate the effect of relative age at entry into school on school performance in Norwegian adolescents.

Of the mental health problems studied, only the SDQ peer problems in boys differed significantly between the three age groups; the scores were highest in the youngest group, although the effect size was small. This might indicate that the youngest pupils in the class experienced difficulty being accepted by their peers. Further studies are required to determine whether the feeling of lack of acceptance among the relatively young pupils influences school performance, and whether boys are more likely to experience peer problems than are girls.

Trends in both emotional subscores in the SDQ and SCL-10 went in the direction opposite to that expected in girls, because girls in the oldest group had higher scores on emotional problems than their younger class mates (although the effect size was small). Our data differ from those of other studies of adolescents, which reported more emotional problems, including self-reported mental health problems, in the youngest age groups [11]. The explanation for these differences might be a more rapid increase in emotional distress in girls than in boys during mid-adolescence [28-30], an effect that may have been more prominent in our older participants (15–16 years) than in the 11–15-year-old pupils studied by Goodman et al. [11].

It is possible that a systematic effect of age at entry into school in our sample was obscured by a stronger age effect. Although the relationship between relative age at entry into school and mental health problems persisted in all age groups in the study by Goodman el al. [11], we believe that this effect wanes with increasing age because the relative age difference in a class becomes less important as the adolescents mature. This might explain why our study showed that relative age had less effect on mental health problems in a sample of 15- and 16-year-old adolescents than in the British study, which examined a wider and younger age range.

Another possible explanation for the discrepancy between our results and those from Great Britain is the differences in the school systems. In the past in Norway, children entered school in their seventh year, but now enter at age six. In Britain, children start school at age four or five. In Norway, there is no formal evaluation of performance before the eighth grade, when the pupils are graded for the first time. In Britain, students are evaluated and choose their course of study earlier than in Norway.

The British school system may place greater demands on pupils than the Norwegian system, at least at earlier ages. This pressure might produce greater differences in mental health status between the younger, less mature pupils and their older classmates in a British setting compared with those in a Norwegian setting [3,9,18]. However, this cannot explain why the relatively older girls in our study reported more emotional distress than their younger peers. One explanation might be the rapid increase in emotional distress among girls during mid-adolescence, as discussed earlier. Another possible explanation is that Norway has a stronger season-of-birth effect than countries located further south, which might manifest as elevated depressive symptom levels among people born during the late winter [8,31]. These hypotheses require further investigation.

Our conclusion about systematic gender differences in the effects of age at entry into school rests on two of 64 possible significant interaction effects (age at entry with each of the covariates for eight outcome measures), and only one was significant at the 0.001 level. We urge caution when drawing conclusions from these relationships.

The strength of our study was the high response rate (88.3%) from all 15- to 16-year-old adolescents in Oslo for two consecutive years in a multicultural environment. Our study might have had selection problems, but these are unlikely to have affected our observations. Two validated questionnaires were used to assess mental health problems and we controlled for known possible confounders in the data analysis.

An important limitation of this study was that all effect measures were based on self-report, and we were unable to include a third-party evaluation by teachers, parents, or diagnostic interviews. Another limitation is that we do not know the extent to which differences in peer or emotional problems affect the average pupil in everyday life, because we did not include the impact part of the SDQ. The study would have benefited by the inclusion of younger age groups to examine whether the effects of relative age within the school year differ between primary school and junior high school, from which our sample was taken.

One of the problems incurred when calculating many main effects and interactions in a single study is the increased risk of identifying significant results by chance. We believe that conclusions should be drawn with care from our data. We were also unable to account for the pupils who did not participate because they were absent temporarily or had dropped out of school. We chose to exclude pupils who had begun school later or earlier than normal, which might have restricted the range of outcome variables somewhat, although we think it unlikely that such a (moderate) restriction in range affected the results.

## Conclusion

Our study shows that relatively young age within a school year has a small but significant effect on school performance in adolescents. The effect of relative age on mental health was weak and significant only for peer problems among boys and emotional problems among girls (opposite to the predicted direction). More studies are required to confirm the relatively weak associations demonstrated between relative age within a school year and school performance and mental health problems. The results from this study do not show effect sizes large enough to justify changes in the way children are enrolled in school in Norway.

## Competing interests

The author(s) declare that they have no competing interests.

## Authors' contributions

LL carried out the statistical analyses and drafted the manuscript. KT took part in the statistical analyses and commented on the drafts. SH and BO took part in writing the methods section and commented on the drafts. EB took part in planning the study, participated in its design and coordination, and commented on the drafts. All authors read and approved the final manuscript.

## Pre-publication history

The pre-publication history for this paper can be accessed here:


